# A Person-Centered Approach to Prison Behavior Based on Officers’ Observations: Relations to Risk, Prison Misconduct, and Recidivism

**DOI:** 10.3389/fpsyt.2020.00241

**Published:** 2020-04-03

**Authors:** Joscha Hausam, Robert J. B. Lehmann, Klaus-Peter Dahle

**Affiliations:** ^1^Institute of Forensic Psychiatry, Charité–Universitätsmedizin Berlin, Berlin, Germany; ^2^Department of Psychology, Medical School Berlin, Berlin, Germany; ^3^Department of Psychology, University of Hildesheim, Hildesheim, Germany

**Keywords:** prison behavior, behavior rating scale, prison officers, risk assessment, prison misconduct, recidivism, treatment evaluation, latent profile analysis

## Abstract

Incorporating measures of prison behavior into risk assessment and management procedures may assist in treatment planning, risk monitoring, and decision-making. A behavior rating scale was used to assess prison officers’ observations on externalizing, internalizing, and adaptive behavior in a sample of 277 sexual and violent offenders in correctional treatment in Berlin, Germany. The present study employed latent profile analysis to identify inmate subtypes with similar behavioral patterns. Results indicated a solution with five latent profiles that showed similarities with previous inmate typologies. The subtypes were termed “Aggressive-Psychopathic,” “Asocial,” “Situational,” “Inconspicuous, and “Inadequate-Dependent.” Analyses attested to the construct and predictive validity of the subtypes and involved the examination of differences on criminological characteristics, risk assessment instruments, various types of prison misconduct, and postrelease recidivism. This person-centered study illustrates the importance of attending to broader patterns of inmate behavior. The structured assessment of behavioral observations by prison officers can be a valuable and easy-to-implement approach to benefit from this largely neglected resource.

## Introduction

Research has led to the development of several generations of risk assessment tools that incorporate static and dynamic risk factors that are theoretically and empirically linked to recidivism (1). In correctional practice, risk assessment is an ongoing task to inform management and treatment efforts, directed at “preventing” rather than “predicting” future risk (2, 3). Lately, greater emphasis has been placed on risk assessment procedures that offer guidance to practitioners in the management and reduction of risk (4). Procedures that incorporate current prison behavior into risk assessment have been proposed to assist treatment planning, risk monitoring, and decision-making (5–7). This person-centered study proposes a feasible approach to identify meaningful subgroups of inmates based on their prison behavior. It can be implemented in daily prison routines at low expenses being based on behavioral ratings by prison officers. Such a classification may be relevant for both research and correctional practice to improve understanding of prison behavior, to match inmates to appropriate treatment, and to predict future offending (8).

### Prison Behavior: Theoretical Background

When explaining prison behavior, researchers have generally relied on three theoretical models. According to the *importation model*, prison behavior is influenced by individual characteristics and preprison experiences such as age, criminal history, and personality. They postulate that prisons are not completely closed systems (9). In contrast, *deprivation models* hold that prison behavior is inflicted by the “pains of imprisonment” and is driven by a lack of goods, services, and liberty (10). Imprisonment represents a serious incision in someone’s life; however, it has been argued that focusing on the adverse effects alone falls short in understanding prison behavior (11, 12). Originating from this approach, the *situational* or *management models* postulate that features of the institutional setting affect prison behavior, such as physical environment, staff resources, and appropriate case management (13). Empirical evidence has generated ample support for these models and integrated models were proposed [for a review see (14)]. The present study is guided mainly by the importation model while keeping in mind that situational factors have an impact on both prison adjustment and misconduct (13). Specifically, the prison environment has the potential to reinforce, alter or suppress behaviors (15).

### Prison Behavior: Empirical Evidence and Conceptual Considerations

Although prison adjustment is a complex experience for inmates, research was largely guided by a focus on problem behaviors that violate social order and safety (12). In accordance with the importation model, several individual characteristics were identified that are related to misconduct and violence in prison, such as age, criminal history, and antisocial attitudes (16–18). Generally, these studies indicate that determinants of prison misconduct and violence are similar to those “that have traditionally provided insight into postrelease recidivism” [(19); p. 710]. Hence, recent life course/developmental perspectives suggest that prison misconduct may rather represent a continuation of a pattern of delinquency (20–22) than an interruption (23). It was emphasized that studying prison behavior can further improve the understanding of recidivism (20).

Research has attested to the predictive validity of prison misconduct in terms of recidivism (24–27). However, Trulson et al. (19) indicated a less clear relationship between misconduct and recidivism in a sample of 1.804 violent offenders in juvenile corrections. Considering different types of prison misconducts (e.g., staff assault, possession of a weapon), they reported that only the total number of misconducts was slightly related to the dichotomous criteria of postrelease arrest. Mooney and Daffern (28) also examined the relationship between official records on aggressive misconduct and recidivism in a sample of 148 offenders, who were predominantly convicted of violent crimes. In terms of predictive validity, they reported a significant, but rather small association with recidivism. In a next step they examined the incremental contribution of aggressive misconduct (e.g., controlling for risk level). The effect diminished and was found only for the subgroup with three or more aggressive incidents. In line with Trulson et al. (19) they concluded that repeated aggressive misconduct is a valuable information to supplement risk assessment procedures. However, they expressed skepticism about official records being a “valid indicator of an ongoing propensity for violence” [(28); p. 325], because they most likely underestimate actual misbehavior. Similarly, Adams [(12), p. 294] stated that “prison disciplinary records clearly are imperfect measures of inmate behavior, being subject to detection and reporting biases”.

Pearson and McDougall (29) pointed out that official records capture only the “tip of the iceberg” of risk-related behavior in prison. Referring to Goldstein (30), they argued that so-called lower-level antisocial behaviors, such as insults, threats, and defiance, are common in prison but are often not communicated by default within risk management procedures (29). Atkinson and Mann (31) conducted a qualitative study examining what types of behavior prison officers observe and subsequently report. They suggested that prison officers are generally experienced observers and identified three factors indicating why some behaviors may not be reported: Habituation (e.g., elevated acceptance towards anti-social behaviors in prison), procedural factors (e.g., not enough time or feedback is ultimately not considered in decision-making), and individual staff factors (e.g., lack of confidence or maintaining rapport with inmates). The authors concluded that “these types of observations could, if utilized appropriately, improve the process of forensic psychological risk assessment; specifically in relation to focusing on current functioning to complement traditional forensic methods which tend to focus on past behavior” [(31); p. 152].

### Prison Behavior: Assessment and Classification

Early attempts to classify inmates according to their prison behavior were undertaken primarily for security reasons. The Adult Internal Management System (AIMS) was developed to assist prison management in dealing effectively with different types of inmates (32). The classification process is based on two checklists completed by prison officers. The life history checklist captures information about the background of an inmate. The prison adjustment checklist includes observations on inmate behavior during the first weeks in prison. Based on the combined scores inmates were classified into one of five subtypes: (a) The *Aggressive-Psychopathic* is described as most aggressive, violent and with little concern for others and having the most trouble with staff). (b) The *Manipulative* type consists of inmates that are less aggressive and confrontational, but no less hostile, untrustworthy, unreliable. (c) The S*ituational* consists of inmates that are generally responsible, trustworthy, and not overly aggressive. They have generally less extensive criminal histories than the first two types. (d) The *Inadequate-Dependent* type appears passive and withdrawn and is rarely involved in prison misconduct. (e) The *Neurotic-Anxious* subtype is anxious, worried, and easily upset. The central objective of the classification system is to separate inmates into housing units by differentiating predators (i.e., the first two types) from their presumed victims (i.e., the last two types). Psychometric properties of the checklists (33, 34) and predictive validity of the AIMS were strongly criticized (35). Nonetheless, construct validity of the typology was supported in a subsequent study (36). Van Voorhis (36) concluded that such a classification approach is promising with regard to treatment planning, since the subtypes showed differential responses to specific treatment interventions.

Behavior rating scales allow a quick and reliable assessment of specific behaviors with many advantages when administered by an observer who is familiar with the subject. In contrast to checklists, they are more suitable to capture gradual characteristics of behavior (37). They provide quantifiable and normative data, which can be used to compare ratings across groups, settings, and time. From a methodological perspective, rating scales improve accuracy of clinical judgement by aggregating clearly operationalized observations (38). Previous research with offenders attested to the reliability and predictive validity of staff rating scales in terms of prison misconduct and violence (39–43). Furthermore, they were used as a means to evaluate the effectiveness of an inpatient violent treatment program (44).

In a similar line of research, Hausam et al. (45, 46) introduced the SWAP-Rating Scale (SWAP-RS) including 40 partly reformulated items of the Shedler-Westen Assessment Procedure-200 [(47); German version: (48)]. The SWAP-200 is an observer-rating tool designed to assess, quantify, and compare clinical observations. It allows for a dimensional assessment of personality and psychopathology in psychiatric (49) and forensic populations (50). Therefore, the items of the SWAP-200 were considered to offer an appropriate framework to systematically assess prison officers’ observations of inmate behavior. The central objective of the scale is to identify, monitor, and communicate behaviors that are relevant to correctional treatment. With reference to the principles of effective offender treatment (51), we intended to include behavioral characteristics that may be indicative of criminogenic needs (e.g., impulsivity), noncriminogenic minor needs (e.g., depression), and strengths (e.g., dependability). Factor analysis revealed an underlying three-factor solution of the SWAP-RS (46), which largely resembles the structure of hierarchical models of psychopathology [e.g., (52)]. Externalizing Prison Behavior (EPB) includes mostly disruptive behaviors directed towards the environment (e.g., hostility, impulsivity). The EPB has found to be most promising in the identification and monitoring of risk-relevant prison behavior. EPB ratings were predictive of prison misconduct and violence as well as violent recidivism after release. Adaptive Prison Behavior (APB) captures features of psychological health, resources, and strengths. APB ratings predicted whether an inmate was granted temporary absence or minimum-security placement. Finally, Internalizing Prison Behavior (IPB) includes behavioral characteristics related to negative emotionality and social withdrawal. Although some significant associations with violent misconduct and recidivism were reported, predictive validity of the IPB was less compelling.

The validation study on the SWAP-RS followed a “variable-centered” approach, largely focusing on specific behaviors and their relationships with outcome variables of interest. However, this approach might not account for the “reality” that these behaviors do not exist in isolation but rather interact. A “person-centered” approach instead focuses on an individual’s overall behavior. By identifying subtypes with similar behavioral patterns, we seek to gain greater insight how the inmate, rather than just his individual behaviors, interacts with the prison environment. In line with previous research on inmate typologies [e.g., (32, 36)], we propose that such an approach may improve our understanding of prison behavior and may have implications for treatment planning and risk assessment (8).

## Purpose of Study

This person-centered study followed three objectives. First, we used Latent Profile Analysis (LPA) in a sample of male sexual and violent offenders to identify prison behavior subtypes. Based on previous research (32, 36) and conceptual considerations, we hypothesized to find four subtypes based on correctional officers’ ratings on the SWAP-RS:

- subtype with high externalizing behaviors (EPB), average/low internalizing and low adaptive behaviors (sensu latiore Quay’s *Aggressive-Psychopathic* type),
- subtype with high EPB, high/average APB and low IPB scores (*Manipulative* type),
- subtype with high APB as well as low EPB and IPB scores (*Situational* type), and
- subtype with high IPB as well as low EPB and APB scores (*Inadequate-Dependent* type).

Since the SWAP-RS does not include characteristics related to fear and anxiety, we did not expect to identify the Neurotic-Anxious subtype. Second, we examined whether the subtypes thus identified differed in meaningful ways from each other with respect to external variables such as criminological characteristics, risk measures, and various types of prison misconduct. We expected to find younger age, more extensive criminal history from subtypes a) and b), highest risk of reoffending and most misconduct from subtypes a) and b), with more violent misconduct expected from subtype a), and lower risk and less prison misconduct from subtype c) as well as d). Third, we examined whether the subtypes differed with respect to recidivism after release from prison. We expected the highest recidivism rates for subtypes a) and b).

## Methods

### Sample

The present study is based on an extended sample of the initial validation study (46). The current sample consisted of *N* = 277 male juvenile and adult inmates in correctional treatment from Berlin (Germany). Specifically, the subsamples were collected from social-therapeutic units for adults (n = 148) and juveniles (n = 75), as well as a preventive detention unit (n = 54). These units generally follow a group-based approach of rehabilitation and encompass a mix of individual and group therapy, social skills training, and educational or vocational training. Apart from therapeutic staff, specifically trained prison officers are part of these units to surveil, supervise, and support prisoners. The offenders of the sample were convicted of sexual offenses (48.9%), violent offenses (47.1%) and other offenses (4.0%). The inmates were convicted to an average sentence of 6.19 years (*SD* = 4.52, Range = 1.50-25^1^). The age at assessment varied from 17 to 82 years (*M* = 37.38, *SD* = 14.54). Most of the inmates were German citizens (79.2%) and had at least on prior conviction (85.1%).

### Procedure

Data was collected between 2014 and 2017 as part of an ongoing evaluation project. The study was carried out in accordance with the recommendations of the Senate for Justice, Consumer Protection and Anti-Discrimination of Berlin, Germany. Ethical approval for the study was sought and granted by the Ethics Committee of Charité— Universitätsmedizin Berlin (EA4/131/18). Prison officers were asked to rate all inmates admitted to one of the three units during that time. Prison officers did not receive a special training in the assessment of the rating scale. A total of 79 prison officers rated on average three inmates (*SD* = 2.36, Range = 1-12) they have known for an average of 18.89 months (*SD* = 22.93, Range = 1-156).

### Measures

#### Prison Behavior

Prison behavior was measured using the SWAP-Rating Scale [SWAP-RS; (46)], a 40-item behavior rating scale with three subscales designed for administration by nonpsychological staff, e.g. prison officers. The SWAP-RS incorporates items of the Shedler-Westen Assessment Procedure-200 [SWAP-200; (47)], an observer-rating tool for personality assessment. The items are written in clear and jargon free language designed to systematically assess and quantify behavioral observations. Of the original 200 statements, the SWAP-RS includes 40 partly reformulated items to assess relevant inmate characteristics and behaviors in prison. Initial item selection process was guided by empirical [i.e., factor loadings; (53)] and conceptual considerations [e.g., appropriateness for prison context; see (46) for item list]. A 5-point Likert type response format corresponds to the frequency of observed behavior (from “never” to “very frequently observed”; scored 0 to 4). The first subscale, EPB, reflects problematic and disruptive behaviors that are directed towards others including psychopathic (e.g., “Appears to experience no remorse for harm or injury caused to others”), narcissistic (e.g., “Has an exaggerated sense of self-importance”), hostile (e.g., “Tends to express intense and inappropriate anger that is out of proportion to the situation at hand”) and emotionally dysregulated features (e.g., “Tends to become irrational when strong emotions are stirred up”). APB consists of a collection of social (e.g., “Is empathic, sensitive and responsive to other peoples’ needs and feelings”) and emotional (e.g., “Is capable of hearing information that is emotionally threatening”) functioning strategies in the prison environment. IPB includes rather inward focused adverse behaviors that are characteristic of schizoid (e.g., “Appears to have little need for human company or contact, is genuinely indifferent to the presence of others”) and dysphoric orientation (e.g., “Tends to feel he is inadequate, inferior, or a failure”). Hausam et al. (46) reported acceptable internal consistencies (average Crohnbach’s alpha = .91) and inter-rater reliability (average ICC = .45) of the SWAP-RS. Subsequent studies found further support for the inter-rater reliability of the measure in a correctional (54 ); mean ICC = .64) and a psychiatric treatment setting [(55); mean ICC = .68]. Noteworthy, in all these studies the prison officers did not receive special training in the assessment of the rating scale.

#### Criminological Characteristics

The following variables were coded based on file review: age at the point of assessment, number of previous convictions, previous prison experience in years, index violent offense (yes/no), and whether the inmate was placed in juvenile prison (yes/no).

#### Risk Assessment

Trained research assistants independent of the correctional treatment facilities completed risk measures according to the German versions of the Level of Service Inventory—Revised [LSI-R; (56)], the Structured Assessment of Protective Factors for Violence Risk [SAPROF; (57)], and the Psychopathy Checklist—Revised [PCL-R; (58)] based on file review. The LSI-R was selected as a measure of general risk of recidivism and the SAPROF as a measure of protective factors, and the PCL-R as measure of the psychopathy construct. The latter is not a risk assessment measure but has shown to be a robust predictor of persistent delinquency. Predictive validity of the measures is well documented, also in German speaking samples [e.g., (59)].

#### Prison Misconduct

A follow-up file review was conducted *M* = 17.69 months (*SD* = 10.71, Range = 3.65–57.33) after the behavioral assessment. Various types of prison misconduct were assessed from files based on disciplinary records. We coded the absence/presence of violence against inmates and staff (e.g., verbal threats, physical assaults), house rule violations (e.g., disturbance during sleeping hours), possession of forbidden objects (e.g., self-made weapon, cell phone), and possession and/or use of drugs. The frequencies of prison misconduct in the total sample were 31.4% (*n* = 87; violence against inmates), 22.0% (*n* = 61; violence against staff), 22.7% (*n* = 63; house rule violations), 50.9% (*n* = 141; possession of forbidden objects), and 26.4% (*n* = 73; possession and/or use of drugs).

#### Recidivism

Postrelease recidivism data for a subsample (*n* = 149) was obtained from police records with an average follow-up of 30.71 months (*SD* = 12.98, Range = 1.31-50.92). These records capture whether the police accused or apprehended a person being a primary suspect of an offense. Therefore, they have a lower threshold compared to convictions based on criminal records. Furthermore, the records only cover crimes committed in Berlin, but not whole Germany. We coded the absence/presence of a non-violent/non-sexual (e.g., thievery, drug offense), violent (e.g., robbery, assault), and sexual (e.g., sexual abuse) future incident that resulted in a police charge. Because of the low rates of sexual incidents (*n* = 6; 4.1%), the latter two were collapsed into one category of severe recidivism. Rates in the sample were 38.5% (*n* = 57) for non-severe (i.e., non-violent/non-sexual recidivism, and 25.7% (*n* = 38) for severe (i.e., violent and/or sexual) recidivism.

### Data Analysis

Latent Profile Analysis (LPA) is a person-centered approach that seeks to identify homogenous subtypes of individuals that share similar characteristics. Statistically, it is similar to Latent Class Analysis but based on observed continuous rather than categorical variables. In the current study, LPA was used to determine whether homogeneous prison behavior subtypes could be captured in a heterogenous sample of male inmates in correctional treatment. Information criteria and likelihood ratio tests were used to identify the optimum number of latent classes. We followed an analytic hierarchy process based on the fit indices BI, AIC, AW, CLC, and KIC (60). We also considered the results of the Bootstrap Likelihood Ratio Test [BLRT; (61)]. The BLRT allows examining whether adding one more latent class significantly improves the model fit. If this is not the case, the more parsimonious model with fewer latent classes should be selected (62). However, the selection and interpretation of a solution should not only be based on statistical criteria, but should also take into consideration model parsimony, simplicity, and clarity (63). For further analyses, the inmates were assigned to the class according to the maximum probability of latent profile membership. According to Clark and Muthén (64), the use of most likely class membership assignment is further justified when entropy is .80 or greater.

Regression analysis was used to examine differential associations of the subtypes with external variables. A regression-oriented approach seemed more favorable than a mean-oriented approach (e.g., analysis of variance) to detect group differences in terms of test power (64). First, multinomial logistic regression analysis was carried out to investigate differences between subtypes on criminological variables and several risk measures. The variables were entered into multinomial regression analysis as covariates to predict class membership. Because the subtypes were compared to each other by varying the reference group to cover all possible comparisons, we controlled for family-wise error by using Bonferroni correction. Second, binary logistic regression was used to predict the probability of each subtype to commit different types of prison misconduct. Class membership was entered as predictor.

Cox proportional hazard regression analyses were then conducted to investigate differences in recidivism between the subtypes recognizing their varying durations of follow-up and controlling for their risk level. Univariate Cox regression models conducted in advance indicated that the LSI-R was the best predictor for both types of recidivism. Therefore, the LSI-R total score was added to the models as a confounding variable to avoid multicollinearity issues. The time variable was time from date of release to first police charge (for recidivists) or time of release to follow-up data collection date (for nonrecidivists). The latter cases are censored. There were no outliers in the sample (according to dfbeta values) and the assumption of proportional hazards was met in all models (according to partial residuals).

LPA was carried out with the tidyLPA package for R version 3.5 (65). The remaining statistical analyses were performed with SPSS version 24.

## Results

### Latent Profile Analysis

Latent Profile Analysis (LPA) was used to determine whether homogenous subtypes with relatively unique SWAP-RS factor profiles can be found in a heterogenous sample of male offenders in correctional treatment. As shown in **Table 1**, the solutions with latent classes (or profiles) fit the data generally better than a unitary solution without latent classes. Following an analytic hierarchy process, based on the fit indices BIC, AIC, AWE, CLC, and KIC, a model with 5 classes fit the data better than the other solutions. The Bootstrap likelihood ratio test also suggested that a five-class solution offers the best model fit, since the transition to a six-class solution did not indicate any improvement.

**Table 1:** Model fit of the latent profile analysis with up to seven latent classes (*N* = 277).

No. of Latent Classes	Log-Likelihood	BIC	AIC	AWE	CLC	KIC	BLRT, p	Entropy	Posterior probability (Min/Max)	
1	−941.72	1,917.19	1,895.44	1,966.93	1,885.44	1,904.44	–	–	–	–
2	−883.68	1,823.60	1,787.36	1,908.43	1,768.76	1,800.36	0.010	0.70	0.84	0.95
3	−878.44	1,835.62	1,784.89	1,954.99	1,758.25	1,801.89	0.099	0.68	0.60	0.91
4	−860.76	1,822.76	1,757.53	1,976.54	1,722.98	1,778.53	0.010	0.73	0.70	0.89
**5**	**−828.54**	**1,780.80**	**1,701.08**	**1,968.94**	**1,658.67**	**1,726.08**	**0.010**	**0.80**	**0.76**	**0.92**
6	−822.77	1,791.76	1,697.54	2,014.42	1,647.11	1,726.54	0.069	0.78	0.60	0.93
7	−821.49	1,811.70	1,702.98	2,068.99	1,644.41	1,735.98	0.683	0.72	0.56	0.93

BIC, Bayesian Information Criterion; AIC, Akaike’s Information Criterion; AWE, Approximate Weight of Evidence Criterion; CLC, Classification Likelihood Criterion; KIC, Kullback Information Criterion; BLRT, Bootstrap Likelihood Ratio Test. Chosen model is highlighted in bold in the table.

In addition to the LPA fit statistics, conceptual considerations also point to this solution. Following the parsimony principle, the solutions with fewer classes were investigated. Regarding the four-class solution, the classes LC1 and LC2 of the five-class solution were collapsed into one class, which led to an extreme increase in variance of the SWAP-RS factor EPB. The further reduction of classes led to even more heterogeneous subgroups, which could no longer be differentiated in a psychologically meaningful manner.

Therefore, the solution with five classes was chosen for interpretation and further analyses. The average posterior membership probabilities of the classes were .85, .94, .89, .89, and .78, respectively. Entropy and the range of posterior probabilities of the classes were substantial (see **Table 1**), suggesting that the five latent classes represent distinguishable variations of the SWAP-RS factors. Subsequently, each inmate was assigned to the class for which his probability was highest, leading to groups that contained 19, 14, 109, 93, and 42 inmates, respectively.

Descriptive statistics of the SWAP-RS for the five latent classes are presented in **Table 2**. As outlined before, values of 0 correspond to the response *never* observed, 1 = *rarely*, 2 = *occasionally*, 3 = *frequently*, and 4 = *very frequently observed*. Group comparisons revealed significant differences with large effects in EPB (*p* < .001), APB, (*p* < .001), and IPB (*p* < .001). Post hoc comparisons using the Hochberg GT2 criterion were predominantly significant at a *p* < .001 level. There were no significant differences on EPB mean scores between LC3 and LC5, on APB between LC1 and LC2, LC1 and LC5, and LC2 and LC5, and on IPB between LC1 and LC2, and LC3 and LC5.

**Table 2:** Descriptive statistics of the Shedler-Westen Assessment Procedure–Rating Scale (SWAP-RS) factors by latent class.

	LC1 (*n* = 19)	LC2 (*n* = 14)	LC3 (*n* = 109)	LC4 (*n* = 93)	LC5 (*n* = 42)	
*SWAP-RS*	*M* (*SD*)	*M* (*SD*)	*M* (*SD*)	*M* (SD)	*M* (*SD*)	*F*-statistic^a^
**EPB**	0.87 (0.36)	3.04 (0.34)	0.52 (0.33)	1.82 (0.42)	0.41 (0.36)	290.04***
**APB**	0.93 (0.52)	0.74 (0.36)	2.23 (0.42)	1.69 (0.45)	1.01 (0.35)	102.96***
**IPB**	2.65 (0.59)	2.12 (0.58)	0.84 (0.50)	1.47 (0.56)	0.84 (0.57)	65.859***

***p < .001. EPB, Externalizing Prison Behavior; APB, Adaptive Prison Behavior; IPB, Internalizing Prison Behavior. ^a^df(4,272).

**Figure 1:**
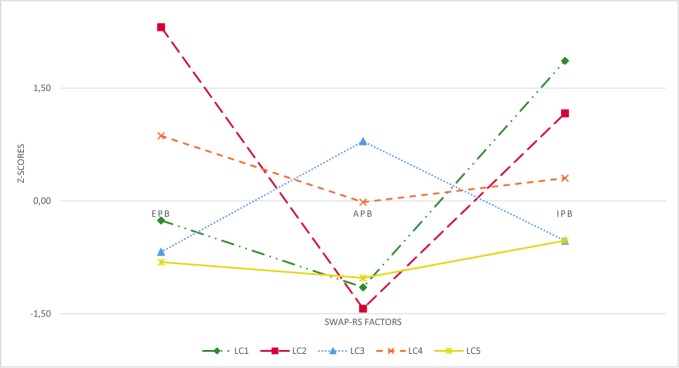
Standardized mean scores of the five latent classes on each Shedler-Westen Assessment Procedure–Rating Scale (SWAP-RS) factor.

For illustration purposes, the SWAP-RS factors scores were transformed to z-scores, with a value of 0 representing the sample mean (see **Figure 1**). Inmates assigned to LC1 (6.9% of the sample) had highest scores on IPB (the mean score indicated: *occasionally* to *frequently* observed), average scores on EPB, and below-average scores on APB (both *rarely* observed). Those allocated to LC2 (5.1%) had highest scores on EPB (*frequently*), below-average scores on APB (*rarely*) and second highest scores on IPB (*occasionally*). In contrast, those allocated to LC3 (39.4%) had below-average scores on EPB and IPB (*never* to *rarely*), but highest scores on APB (*occasionally*). Inmates assigned to LC4 (33.6%) had above-average scores on EPB, whereas scores on APB and IPB were both average (absolute average values indicated *rarely* to *occasionally* observed) Finally, those allocated to LC5 (15.2%) scored below-average on all three factors (*never* to *rarely* observed).

### Relations With External Variables

#### Criminological Characteristics

**Table 3** summarizes the criminological features by class. Only minor differences in age were observed. Inmates assigned to LC1 were oldest (*M* = 44.37, *SD* = 13.02; Median = 47.95; Range = 17 - 66), but significantly different only from inmates assigned to LC4 (*M* = 35.34, *SD* = 35.35). In terms of previous convictions there were some differences, with inmates assigned to LC3 (*M* = 4.61, *SD* = 4.27) having the least and inmates assigned to LC5 (*M* = 7.60, *SD* = 7.40) having the most previous convictions. The differences were statistically significant between LC3 and LC1 (*M* = 7.00, *SD* = 6.68), LC4 (*M* = 6.68, *SD* = 5.32), and LC5 (*M* = 7.60, *SD* = 7.60). No significant differences were observed on past prison experience. Similarly, the proportion of violent index offense and placement in juvenile prison was evenly distributed across classes (see **Table 3**).

**Table 3:** Criminological characteristics by latent class.

	LC1 (*n* = 19)	LC2 (*n* = 14)	LC3 (*n* = 109)	LC4 (*n* = 93)	LC5 (*n* = 42)
	*M (SD)*	*M (SD)*	*M (SD)*	*M (SD)*	*M (SD)*
Age	44.37 (13.02)_a_	36.31 (15.37)	38.03 (15.22)	35.35 (12.73)_a_	37.59 (16.51)
Previous convictions	7.00 (6.68)_a_	6.57 (6.17)	4.61 (4.85)_a,b,c_	6.68 (5.32)_b_	7.60 (7.40)_c_
Past prison experience (years)	4.93 (6.31)	5.26 (7.12)	4.27 (7.32)	4.42 (5.64)	5.39 (6.44)
	% (*n*)	% (*n*)	% (*n*)	% (*n*)	% (*n*)
Violent index offense (*n*=130)	42.1 (8)	42.9 (6)	45.0 (49)	46.7 (43)	57.1 (24)
Juvenile (*n*=75)	5.3 (1)	35.7 (5)	29.4 (32)	28.0 (26)	26.2 (11)

Subscripts denote significant differences between classes in multinomial logistic regression models after Bonferroni correction.

#### Risk Assessment

**Table 4** contains the total scores of the risk assessment instruments across classes. Multinomial regression analyses indicated the clearest trend for inmates assigned to LC2, having the highest average scores on the LSI-R (*M* = 29.15, *SD* = 6.15) and PCL-R (*M* = 20.33, *SD* = 6.40) as well as the lowest score on the SAPROF (*M* = 10.54, *SD* = 3.50). These values were largely different from the inmates assigned to LC3 and LC5, but not from LC1 and LC4. For inmates assigned to LC4 a similar but less pronounced picture emerged with regard to the total scores of the LSI-R (*M* = 27.37, *SD* = 6.62), PCL-R (*M* = 17.27), and SAPROF (*M* = 12.82, *SD* = 3.60). Some significant differences were found compared to inmates assigned to LC3 and LC5. While the inmates assigned to LC1 showed a risk profile similar to those assigned to LC2 and LC4, the scores of the LSI-R (*M* = 27.16, *SD* = 8.89), PCL-R (*M* = 16.12, *SD* = 8.33), and SAPROF (*M* = 12.26) did not differ significantly from any other class. As stated above, lowest LSI-R and PCL-RS as well as highest SAPROF scores were observed for inmates assigned to LC3 and LC5.

**Table 4:** Risk measures by latent class.

	LC1 (*n* = 19)	LC2 (*n* = 14)	LC3 (*n* = 109)	LC4 (*n* = 93)	LC5 (*n* = 42)
	*M* (*SD*)	*M* (*SD*)	*M* (*SD*)	*M* (*SD*)	*M* (*SD*)
**LSI-R**	27.16 (8.89)	29.15 (6.14)_a,b_	24.04 (6.72)_a,c_	27.37 (6.62)_c,d_	23.20 (8.77)_b,d_
**SAPROF**	12.26 (4.64)	10.54 (3.50)_a,b_	13.82 (3.97)_a_	12.82 (3.60)_c_	14.55 (4.26)_b,c_
**PCL-R**	16.12 (8.33)	20.33 (6.40)_a_	14.56 (5.95)_a,b_	17.27 (6.38)_b_	15.96 (7.77)

LSI-R, Level of Service Inventory – Revised; SAPROF, Structured Assessment of Protective Factors for violence risk; PCL-R, Psychopathy Checklist – Revised. Subscripts denote significant differences between classes in multinomial logistic regression models after Bonferroni correction.

#### Prison Misconduct

**Figure 2:**
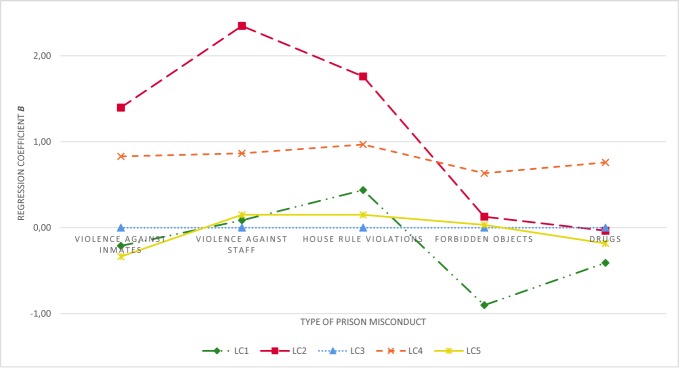
Regression coefficients (*B*) for several types of prison misconduct by Latent Class. LC3 (*n* = 109) served as reference group.

**Table 5** summarizes frequencies of the different types of prison misconduct across classes. Using logistic regression analysis, differences between classes were examined. The dichotomous prison misconduct criterium was predicted by class membership. The inmates assigned to LC3 were selected as reference group based on conceptual considerations and because they represented the largest class. **Figure 2** illustrates the prison misconduct profiles based on the regression coefficient *B* of the inmates assigned to LC1, LC2, LC4, LC5, compared to LC3 (they represent the baseline at 0).

**Table 5:** Types of prison misconduct by latent class.

	LC1 (*n* = 19)	LC2 (*n* = 14)	LC3 (*n* = 109)	LC4 (*n* = 93)	LC5 (*n* = 42)
	% (*n*)	% (*n*)	% (*n*)	% (*n*)	% (*n*)
*Violence against inmates*	21.1 (4)	57.1 (8)	24.8 (27)	43.0 (40)	19.0 (8)
*Violence against staff*	15.8 (3)	64.3 (9)	14.7 (16)	29.0 (27)	16.7 (7)
*House rule violations*	21.1 (4)	50.0 (7)	14.7 (16)	31.2 (29)	16.7 (7)
*Forbidden objects*	26.3 (5)	50.0 (7)	46.8 (51)	62.4 (58)	47.6 (20)
*Drugs*	15.8 (3)	21.4 (3)	22.0 (24)	37.6 (35)	19.0 (8)

Logistic regression analyses revealed a clear trend for inmates assigned to LC2 to be at highest risk to be disciplined for misconduct with regarding violent behavior against inmates (57.1%; *B* = 1.40, *p* < .05) and staff (64.3%; *B* = 2.35, *p* < .001), as well as house rule violations (50.0%; *B* = 1.76, *p* < .01). However, no significant differences were found for the possession of forbidden objects (50.0%) and drugs (21.4%) compared to the reference group LC3. Noteworthy, frequencies for forbidden objects were equally high across classes (50.9%), except for LC1.

The analyses revealed a similar but less pronounced pattern for inmates assigned to LC4 in terms of violence against inmates (43.0%; *B* = 0.83, *p* < .01) and staff (29.0%; *B* = 0.87, *p* < .05) as well as house rule violations (31.2%; *B* = 0.97, *p* < .01). In addition, they were at higher risk to be disciplined for the possession of forbidden objects (62.5%; *B* = 0.63, *p* < .05) and the use or possession of drugs (37.6%; *B* = 0.76, *p* < .05), compared to LC3. No differences in any type of prison misconduct were observed for inmates assigned to LC1 and LC5, compared to inmates allocated to LC3.

#### Recidivism

In a subsample (*n* = 147) Cox proportional hazard regression analyses were conducted to investigate hazards of recidivism of the classes recognizing their varying durations of follow-up. Analyses included *n* = 9 inmates assigned to LC1 (47% of the initial class), *n* = 5 assigned to LC2 (36%), *n* = 64 inmates assigned to LC3 (59%), *n* = 53 inmates assigned to LC4 (57%), and *n* = 17 inmates assigned to LC5 (41%). Follow-up duration did not differ significantly between groups, *F*(4,143) = 1.38, *p* = .243). Nonsevere (i.e., non-violent/non-sexual) recidivism rates were as follows: 33.3% (LC1), 60.0% (LC2), 31.3% (LC3), 43.4% (LC4), and 47.1% (LC5). Severe (i.e., violent and/or sexual) recidivism rates were: 0% (LC1), 60.0% (LC2), 17.2% (LC3), 39.6% (LC4), and 17.6% (LC5). The LSI-R was added to the models as confounding variable. Again, inmates assigned to LC3 were set as reference group.

The Cox regression model predicting non-severe recidivism marginally failed to reach significance, LR-*X*²(5) = 10.73, *p* = .057. As shown in **Table 6** there are no differences between the classes’ hazard ratios. The hazard ratio of the LSI-R significantly differed from 0 (HR = 1.05, *p* < .05).

**Table 6:** Cox proportional hazard regression analyses predicting non-severe and severe recidivism by Latent Class.

	Non-severe recidivism			Severe recidivism		
	*B* (*SE*)	HR	95% CI	*B* (*SE*)	HR	95% CI
LSI-R	0.05 (0.02)	1.05*	1.01–1.09	0.04 (0.03)	1.04	0.99−1.09
*Latent Class*						
- LC1 (*n* = 9)	0.55 (0.62)	1.73	0.51–5.85	−11,91 (351.06)	0	0^b^
- LC2 (*n* = 5)	0.94 (0.63)	2.55	0.75–8.69	1.83 (0.66)	6.22**	1.71−22.66
- LC3 (*n* = 64)^a^						
- LC4 (*n* = 53)	0.41 (0.31)	1.51	0.81–2.78	1,00 (0.38)	2.73**	1.29−5.77
- LC5 (*n* = 17)	0.52 (0.42)	1.69	0.74–3.86	0,06 (0.66)	1.06	0.29−3.84

**p < .01; *p < .05. HR, hazard ratio; SE, standard error; CI, confidence interval. ^a^ reference group. ^b^ The model showed an infinite upper confidence interval value for LC1, since none of the inmates assigned to this class recidivated during the follow-up period.

**Figure 3:**
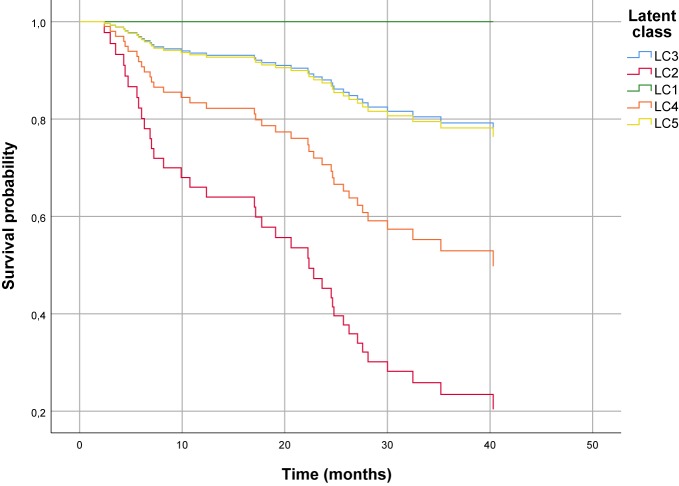
Survival function using Cox proportional hazard regression analysis to predict severe recidivism.

The Cox regression model predicting severe recidivism was found to be significant, LR-*X*²(5) = 22.32, *p* < .001. As shown in **Table 6**, the hazard of severe recidivism was six times higher for LC2 (HR = 6.22, *p* < .01) and almost three times higher for LC4 (HR = 3.08, *p* < .01), compared to LC3. The HRs of the inmates assigned to LC1 and LC5 were not significantly different from 1. The LSI-R remained nonsignificant (HR = 1.04, *p* = .100). **Figure 3** illustrates the survival function by Latent Class of the Cox regression analysis predicting severe recidivism.

## Discussion

This person-centered study sought to identify, describe, and validate behavior subtypes of adult and juvenile offenders in correctional treatment. We examined whether these subtypes varied on criminological characteristics, risk of reoffending, prison misconduct and recidivism. LPA discerned five latent classes (or profiles) based on prison officers’ behavioral observations as measured with the SWAP-RS. Given the high average posterior probabilities of the latent classes, inmates could be assigned to one of the five subtypes with good accuracy. The subtypes showed strong conceptual and empirical similarities with previous research on inmate typologies (32, 36). We mostly followed the descriptive labels of Quay (32), in cases of conceptual deviations we also referred to the results of Van Voorhis (36). The five subtypes are hereinafter labeled as “Inadequate-Dependent” (LC1), “Aggressive-Psychopathic” (LC2), “Situational” (LC3), “Asocial” (LC4), and “Inconspicuous” (LC5). Whereas the first four subtypes largely corresponded to our hypotheses, the finding of the latter was unexpected and requires special consideration. Levinson (33) has proposed to collapse the five subtypes of the AIMS (32) into three broader domains (i.e., Heavies, Moderates, and Lights) to guide the separation of inmates into housing units. Since the subtypes are characterized by distinct behavioral profiles and a classification with regard to internal placement was not an objective of the study, the aggregation of the subtypes is not meaningful in the present study. To facilitate comparison to precedent findings, an aggregation based on conceptual considerations will be used where necessary to highlight differences between and compare distinctive features within these domains. The Aggressive-Psychopathics and Asocials are therefore referred to as Heavies, the Situationals and the Inconspicuous as Moderates and the Inadequate-Dependents as Lights.

The Aggressive-Psychopathic subtype represented the smallest group of inmates (5%). Their most distinctive feature was an increased level of EPB, accompanied by low adaptive (APB) and high internalizing behavior (IPB). In line with Quay (32), these inmates may be described as most aggressive and violent with little concern for others. It has been suggested that the Aggressive-Psychopathic’s potential for violent and explosive behavior is linked to poor emotional control and interpersonal problems with others (66). This finding may correspond to their concurrent high IPB, since the factor includes observations such as lack of relationships and perception as outcasts (46). The leading features of the Asocial subtype were an elevated EPB and average APB (both reflecting second highest ratings in the sample). Asocials were the second largest subgroup, accounting for one third of the sample (34%). As expected, the behavioral profile has strong similarities with the Manipulative (32) or Asocial subtype (36). We have chosen the latter label because EPB covers a wider range of disruptive behaviors as outlined above.

The Situationals were the largest group (39%) in the present sample with highest APB and simultaneously very low EPB and IPB. As expected, they resemble Quay’s type (32) that has consistently replicated in subsequent research (36). Accordingly, this type may be described as cooperative, responsible and trustworthy, with prosocial values, and few problems in prison and conflicts with staff (32). The Inconspicuous subtype was identified contrary to our expectations and formed the third largest group of inmates (15%). As the label shall imply, the leading feature of this subtype was their rating below average on all behaviors. The behavioral pattern indicates that these inmates don’t seem to attract much attention with their behavior, neither in the good nor in the bad, so to say they’re “moving under the radar”. Quay (32) stated that some Moderates try to serve their time as quietly as possible to ensure prompt return to society (32).

Finally, the Inadequate-Dependent subtype represented a minority of inmates (7%) whose outstanding feature was an increased level of IPB, accompanied by low adaptive (APB) and externalizing behaviors (EPB). As hypothesized, the behavioral pattern largely resembles Quay’s (1984) type. The Inadequate-Dependents may therefore be described as socially withdrawn, passive, broody, and joyless.

Subsequently, we examined construct validity of the subtypes by testing the differential associations with theoretically and empirically relevant external variables. Only few differences between subtypes were found in criminological characteristics. As expected, the Situationals showed the fewest previous convictions. Virtually no differences were found between the subtypes regarding criminal history, previous prison experience and violent index offense. This seems surprising at first. Research reported significant associations between prison misconduct and criminal records (17), previous imprisonments (67), and violent index offense (68). Hence, we expected a clearer exposure to these criminological characteristics for subtypes associated with increased EPB (46). Adams (12) noted that these criminological characteristics are more specifically related to prison misconduct, but not to prison adjustment in general. As our person-centered study examines a broader operationalization of prison behavior (i.e., EPB, APB, and IPB) and their interactions, these associations may be blurred and therefore the expected relations were not detectable.

Surprisingly, the subtypes were also largely independent of age. An inverse relationship between age and prison misconduct (17, 18) as well as adjustment problems (69, 70) is one of the most consistently reported findings. For instance, younger inmates tend to act out and resolve their conflicts “in ways that are demonstrably visible and that advertise toughness and strength” [(12); p. 302]. It was therefore expected that younger inmates would be more likely to be found among the subtypes with higher EPB and lower APB ratings. The results provide little evidence for this relationship. An explanation could be that the sample with both adults and juveniles was too heterogeneous to identify such differences. Recent research pointed out that prison behavior may better be examined for juveniles and adults separately (26).

The presence of subtypes, and hence, heterogeneity in the sample, was more evident when differences were examined in relation to risk assessment instruments (i.e., LSI-R, SAPROF) and the PCL-R. The Aggressive-Psychopathics and Asocials showed significantly higher risks of reoffending. For instance, according to the German manual of the LSI-R (56), the Heavies were on average at the upper end of the moderate risk category, whereas the Moderates were at the lower end. Similarly, the Heavies scored consistently higher on the PCL-R and lower on the SAPROF than the Moderates. As the label denotes, Quay (32) proposed that Aggressive-Psychopathics may also be characterized in terms of the psychopathy construct. This was partially confirmed in the present study. The Aggressive-Psychopathics displayed the highest PCL-R scores. They were significantly higher than those of the Moderates. However, most of the Aggressive-Psychopathics did not exceed the suggested threshold for a psychopathy diagnosis [e.g., 25 points in Germany; (71)]. It should be noted that PCL-R ratings were based on file review only, which can result in lower PCL-R scores compared to the standard assessment procedure (72). However, the results may indicate that these inmates rather represent a specific psychopathy subtype (73). For instance, research has reported some evidence for associations between the behavioral features of psychopathy (i.e., factor 2 of the PCL-R) and internalizing psychopathology (74). On the basis of the available data, this must remain a speculation and requires further examination. In summary, the findings lend some support of the construct validity of the subtypes.

Prison misconduct is commonly used as a risk marker in research and correctional practice. We used several types of prison misconduct to examine whether differential patterns could be identified for the subtypes. As expected, the Heavies displayed increased rates of prison misconduct. Noteworthy, we found a distinguishing feature between the two subtypes. The Aggressive-Psychopathics were predominantly sanctioned for outwardly aggressive behavior (i.e., violence against inmates and staff as well as house rule violations). Asocials showed moderate to high rates of any prison misconduct, but less violent misconduct compared to the Aggressive-Psychopathic. In line with Levinson (33), this suggests that the Aggressive-Psychopathics are not only constantly violating rules but are also particularly aggressive with little concern for others. Accordingly, the Asocials seem to be less aggressive and confrontational, but no less antisocial, manipulative, and hostile. More than one third of the Asocials were disciplined for drug use or possession, underpinning their antisocial orientation (51). Accordingly, this subtype was referred to as “committed criminal” elsewhere (66), who exhibits antisocial values with an extensive criminal history and many criminal peers. Also, in agreement with Levinson (33), the Inadequate-Dependents were rarely involved in any kind of prison misconduct (on average only one in five inmates). Both the Situationals and the Inconspicuous showed some, but seldomly violent misconduct in prison.

In a last step, we examined whether certain subtypes are more likely to reoffend after release from prison (i.e., predictive validity). Building on the study by Hausam et al. (46), the aim was to determine whether a person-centered approach may further improve prediction by incorporating a broader range of prison behavior. With regard to non-severe recidivism, the subtypes did not differ significantly from each other. In contrast, severe recidivism did vary as a function of the subtype. In line with Levinson (33), the Heavies had generally the highest recidivism rates. Specifically, the Aggressive-Psychopathics had an estimated probability of severe recidivism six times higher than the Situationals, while the Asocials showed a threefold increase. In terms of numbers, 60% of the Aggressive-Psychopathics and 40% of the Asocials reoffended, while the rate for Moderates was around 17% each. Noteworthy, none of the Inadequate-Dependents reoffended.

Our findings illustrate the use of a person-centered approach as an adjunct to variable-centered research on prison behavior. The explication of the subtypes illustrated some important distinctions that may characterize these subgroups and highlights otherwise hidden diversity. The current findings may have implications for treatment planning and evaluation. Van Voorhis et al. (75) provided evidence that the effectiveness of a cognitive-behavioral skills program varied depending on the personality style of parolees. Using the self-report Jesness-Inventory (76), which classifies offenders into subtypes that are similar to those described above, they reported that the program was most effective for dependent parolees. In contrast, an adverse effect of the program was indicated for neurotic parolees resulting in a higher recidivism rate compared to an untreated control group of the same personality subtype (75).

The SWAP-RS could be utilized in a similar way to address the specific responsivity principle, which is seldomly incorporated into correctional practice or research (77). Basically, the principle maintains that treatment should be tailored to an offender’s learning style, abilities, personality, and motivation. The assignment of subtypes to programs with different methodological approaches may increase the effectiveness of treatment. For instance, a rather confrontational intervention may be more appropriate for Asocials than for Aggressive-Psychopathics (66). In addition, Hausam and Dahle (77) examined changes in prison behavior with regard to the specific responsivity principle of effective offender rehabilitation (51). They assessed the SWAP-RS as well as self-reports on attitudes towards treatment (79) and treatment readiness (80) on two occasions within a year in a sample of *N* = 58 adult offenders in correctional treatment. Using reliable change indices [RCI; (81)], they reported that motivational improvements were significantly associated with reductions in externalizing and internalizing behaviors (78). The SWAP-RS may provide a useful tool in the evaluation of treatment efforts and behavioral changes would be reflected by reclassification.

The results may also have implications for risk assessment and management. Research has attested to the predictive validity of prison misconduct in terms of future recidivism (25–27). However, as described at the outset, official records of prison misconduct are biased measures that capture only the “tip of the iceberg” of risk-related behavior (28). Systematic observations in the prison environment may be an appropriate means to capture lower-level behaviors that may not otherwise be reported. By incorporating a set of behaviors described above (i.e., EPB, APB, and IPB), the SWAP-RS may assist practitioners to identify and monitor behaviors that are related to an inmate’s dynamic risk and protective factors. As indicated by Hausam und Dahle (78), the SWAP-RS is potentially change-sensitive. Periodically reassessed, it could provide a means of monitoring behavioral changes during treatment. For instance, a reduction of disruptive (i.e., EPB) and an increase of prosocial behaviors (i.e., APB) may serve as an indicator of treatment progress. The results of the present study have indicated that the joint consideration may further improve understanding of the interplay of these behaviors.

The implementation of behavior rating scales designed for administration by nonpsychological staff (e.g. prison officers) might help to enhance the status and increase the value of these professions, which might in turn result in higher job satisfaction. Here, the proposed scales constitute a means to address the points raised by Atkinson and Mann (31). Using this framework may help to interrupt the habituation process as it assists prison officers to identify, monitor, and communicate behavior that is not appropriate (e.g., EPB). In terms of the procedural factors, the application of behavior rating scales allows a quick and reliable assessment. The task should be manageable even though there is often not enough time in work routine. Furthermore, the staff will get feedback by including their ratings in decision-making (e.g., monitoring behavioral changes during treatment).

### Limitations and Future Directions

Several limitations of the present study merit consideration. Two out of five clusters (arguably the most extreme ones) consisted of less than 20 individuals each. Although construct and predictive validity of the identified subtypes were consistent with previous inmate typologies, this may raise concerns about the stability of the findings and the likelihood of replication in another sample. Furthermore, the variables in the present study were predominantly risk markers. Additional external variables should be considered in future studies to ensure construct validity of the subtypes that are relevant to correctional treatment (e.g., mental health variables).

Although LPA is a statistical method that is intended to capture heterogeneity in a population, it should be noted that the joint consideration of adult and juvenile offenders might have affected findings. There is evidence that juveniles and adults differ in their behavior in prison (12, 26). Although research indicates that the types described above could be replicated quite consistently in juvenile and adult samples (36), differential patterns may have been masked by the joint evaluation.

The recidivism criteria were obtained on the basis of police records and are likely to be biased. Not all offenses for which offenders are accused or arrested by the police result in charges or convictions. They merely serve as an indicator of future reoffending after release from prison. In addition, data on recidivism were only available for a smaller subsample. This led to a further reduction of the already small number in certain subtypes. Accordingly, the findings should be interpreted with caution. Future research should replicate the current results using a larger sample and another outcome measure of recidivism (e.g., official criminal records).

A conceptual limitation of the approach should be highlighted. As described at the outset, prison behavior is influenced by both individual and situational characteristics. The SWAP-RS solely captures observations on the appearance of behavior, irrespectively of environmental influences. Therefore, future research should include environmental variables (e.g., prison climate) to examine their influences on prison behavior.

Although the Moderates could be distinguished from the other subtypes, only criminal history distinguished the Situationals from the Inconspicuous. Apart from that, they were mostly similar on the examined characteristics. To ensure construct validity of the Situationals and Inconspicuous, the key question is whether these two subtypes differ on anything other than their SWAP-RS profiles that is of relevance. Future studies should investigate two of presumably many possible explanations. First, they may differ in judgeability. According to Colvin (82) judgeable persons are “those who are open and knowable versus those who are closed and enigmatic” (p. 861). Judgeability is considered a stable individual difference linked to a variety of characteristics (e.g., nonverbal communication, extraversion) and plays an important role on how someone is perceived by others (83). Being accurately perceived has, among other things, an impact on person-environment fit, social support, and self-disclosure (83). There is so far no research on judgeability in prison. However, it would certainly be interesting to examine whether judgeability has an impact on treatment or decision-making. Second, the Inconspicuous may be a methodological artifact resulting from systematic ratings by prison officers. In the following the response bias is described in distinction to the assessor’s bias, which is largely influenced by individual information processing (84). The latter could also be of great importance (e.g., leniency or severity effects), but would most likely not cause the potential artifact described above. Given the predominantly low behavioral ratings, the extreme response bias appears to be most relevant (i.e., the prison officer systematically selects the never observed response). Research has indicated relationships between extreme response bias and intelligence/education (85) and personality attributes [e.g., rigidity; (86)]. From a statistical point of view, such systematic responses would affect LPA model estimation. However, in light of the presented construct validity and the large number of different raters in the present study (79 prison officers), it seems rather unlikely but requires further investigation in future research.

Despite these limitations, this study supports the use of a person-centered approach to identify meaningful subgroups of offenders based on their prison behavior. Such an approach allows a more comprehensive understanding of the interactions between specific behaviors. In line with previous research, systematic observations of current prison behavior may provide a valuable source of information for risk assessment, treatment and evaluation. In practice, the described approach can be implemented into daily work routine at low expenses and assists prison officers in the communication of their experiences with inmates. It may also raise staff awareness of lower-level behaviors that otherwise would not be reported.

## Data Availability Statement

The datasets generated for this study will not be made publicly available. This study is part of an evaluation project commissioned by the Berlin Senate for Justice, Consumer Protection and Anti-Discrimination. We do not have the right to disclose the data.

## Ethics Statement

The study was carried out in accordance with the recommendations of the Senate for Justice, Consumer Protection and Anti-Discrimination of Berlin, Germany. Ethical approval for the study was sought and granted by the Ethics Committee of Charité—Universitätsmedizin Berlin (EA4/131/18). Written informed consent from the participants' legal guardian/next of kin was not required to participate in this study in accordance with the national legislation and the institutional requirements.

## Author Contributions

JH conceived of the present study, performed statistical analyses, and wrote the first draft of the manuscript. RL revised the first draft. K-PD supervised the project. All authors have contributed to the manuscript and agreed to authorship in the indicated order.

## Funding

The author(s) disclosed receipt of the following financial support for the research, authorship, and/or publication of this article: The evaluation project was funded by the Senate for Justice, Consumer Protection and Anti-Discrimination of Berlin, Germany

## Conflict of Interest

The authors declare that the research was conducted in the absence of any commercial or financial relationships that could be construed as a potential conflict of interest.

## Footnotes

1 Eight offenders served a life sentence. In line with the International Criminal Court in the Hague, Netherlands, life sentences were generally coded as 25 years. In 2015, 59 offenders serving a life sentence in Germany were released after 19 years.
